# Oral Microbiome and Constipation: A Causal Link Revealed by Mendelian Randomization

**DOI:** 10.1002/jgh3.70390

**Published:** 2026-03-25

**Authors:** Ziyi Zhao, Jinlian Ling, Jiashu Chen

**Affiliations:** ^1^ Faculty of Biology, Medicine and Health University of Manchester UK; ^2^ Jiangxi University of Chinese Medicine Jiangxi China

**Keywords:** constipation, gut microbiota, Mendelian randomization, oral microbiome, oral‐gut axis

## Abstract

**Background:**

Constipation affects approximately 15.3% of the global population. While the gut microbiome's role in constipation has been studied, the causal relationship between the oral microbiome and constipation remains unexplored.

**Methods:**

We utilized Mendelian randomization (MR) and large‐scale GWAS data to investigate the causal relationship between the oral microbiome and constipation. Oral microbiome data were sourced from a metagenome‐wide association study (mgGWAS) on 2984 individuals, while constipation GWAS data came from 176 629 samples in the Japan Biobank. Statistical methods included inverse variance‐weighted (IVW) analysis, weighted median, and MR‐Egger regression.

**Results:**

The MR analysis revealed significant associations between specific oral microbiome and constipation. *Treponema denticola* , found in saliva, was positively associated with an increased risk of constipation (OR = 3.961, 95% CI = 1.085–14.453, *p* = 0.037). Conversely, certain bacteria like *Pauljensenia* sp000308055 showed protective effects (OR = 0.409, 95% CI = 0.167–0.999, *p* = 0.0496). In the tongue coating, *Neisseria sicca* exhibited a significant positive association with constipation (OR = 4.864, 95% CI = 1.293–18.302, *p* = 0.019), while *Aggregatibacter segnis* demonstrated a protective effect (OR = 0.400, 95% CI = 0.188–0.854, *p* = 0.018).

**Conclusion:**

This study is the first to explore the potential causal relationship between oral microbiome and constipation. The findings suggest that specific oral bacteria may influence the risk of constipation, highlighting the need for further research to validate these relationships and understand the mechanisms involved. Moreover, the study underscores the importance of considering both oral and gut microbiome in the context of gastrointestinal health and disease management.

## 1 Introduction

Constipation is a common gastrointestinal disorder characterized by infrequent bowel movements, hard or lumpy stools, and difficulty in defecation. According to statistics, approximately 15.3% of the population suffers from constipation worldwide [1]. Constipation is not life‐threatening, but it can significantly impact the quality of life and lead to complications such as hemorrhoids, anal fissures, and fecal impaction if left untreated [2, 3]. Understanding the etiology and pathophysiology of constipation is crucial for developing effective treatment strategies.

Recent studies have suggested that the human microbiome might play vital roles in treating constipation. A RCT showed that the probiotic formula with *Bifidobacterium animalis* subsp. *lactis* HN019 + *Lacticaseibacillus rhamnosus* HN001 effectively relieved hard stool in functional constipation patients after 4 weeks intervention [4], another study identified that gut microbiome *Coprococcus* and *Bacteroidetes* were causally associated with constipation [5]. These studies primarily focus on the relationship between the gut microbiome and constipation. However, the relationship between the oral microbiome residing in the upper digestive tract and constipation has been seldom studied.

The oral microbiome, residing in the human oral cavity, is highly diverse, encompassing over 700 bacterial species and interacting with the human host's immune system. These microorganisms are not only associated with oral health but are also believed to be closely linked to Alzheimer's disease, diabetes, cardiovascular diseases, and various cancers [6]. Despite residing in the oral cavity, numerous studies have demonstrated a connection between the oral microbiome and lower gastrointestinal diseases. It has previously been reported that specific oral bacteria, such as a subset of *Porphyromonas gingivalis* , *Streptococcus mutans* , and *Fusobacterium nucleatum* , *Campylobacter concisus* , may exacerbate inflammation in Crohn's disease [7]. Additionally, four distinct species or phylotypes of *Staphylococcus* and other organisms of the oral microbiome were found exclusively in patients with ulcerative colitis and were undetectable in healthy individuals [8]. *Fusobacterium periodonticum* has been associated with colon cancer, and the study of the relationship between the oral microbiome and colorectal cancer is considered highly valuable [9].

As gut microbiome continues to be extensively studied [10], an increasing number of researchers believe that both oral and gut microbiome should be considered together in their impact on diseases. The oral microbiome is thought to influence gut microbiome through three main pathways: the enteral route, the hematogenous route, and immune cell migration. Conversely, changes in the abundance of gut microbiome can also affect the oral microbiome. Research on the interactions between oral and gut microbiome is still in its early stages, and the implications of these interactions for the development and progression of various diseases remain unclear. Therefore, studying the mechanisms that maintain and regulate the balance of oral and gut microbiome is essential for understanding the onset and progression of diseases [11].

Mendelian randomization (MR) studies, as a novel statistical approach, use single nucleotide polymorphisms (SNPs) as instrumental variables (IVs) to explore causal relationships between exposures and outcomes [12]. This method has been widely used to investigate the causal relationships between gut microbiome, oral microbiome, and various diseases [13, 14]. Previous research has explored the relationship between gut microbiome and constipation [5]. To further understand the impact of oral‐gut microbiome on constipation and identify more potential therapeutic targets, this study employs MR to investigate the causal relationship between oral microbiome and constipation.

## 2 Methods and Materials

### 2.1 Data Sources

Oral microbiome data was derived from a large‐scale metagenome‐wide association study (mgGWAS), which was conducted on tongue dorsum samples from 2984 healthy Chinese individuals and saliva samples from 1915 individuals collected in 2017, providing high‐depth whole‐genome sequencing data. Furthermore, the identified associations were validated in an independent replication cohort of 1494 individuals, who also had metagenomic sequencing data and relatively low‐depth whole‐genome sequencing data. Numerous consistent associations were discovered between genetic loci and the microbiome of both the tongue dorsum and saliva [15].

GWAS data for constipation were derived from data from 220 deeply phenotyped GWAS conducted by the Japan Biobank. High‐quality genetic linkage group data from East Asian populations. This large‐scale non‐European population genetic linkage map was selected for this study. A total of 176 629 constipation data samples were collected, and a total of 12 454 459 SNPs were included. An annotated version was performed using HG19/GRCh37 after removing linkage imbalances and adjusting for sex, year of birth, genotyping batch, and the first four principal components [16]. The GWAS characteristics of the original data source are shown in Table 1.

**TABLE 1:** Summary of the GWAS included in this MR study.

Phenotype	Consortium	Ethnicity	Sample sizes	Number of SNPs	Year
Oral microbiome	CNGBdb	East Asian	2948 (Tongue *N* = 2017, Saliva *N* = 1914)	16 432 (Tongue *N* = 8426, Saliva *N* = 8009)	2021
Constipation	EBI	East Asian	176 629	12 454 459	2021

### 2.2 Study Design and IVs

**FIGURE 1:**
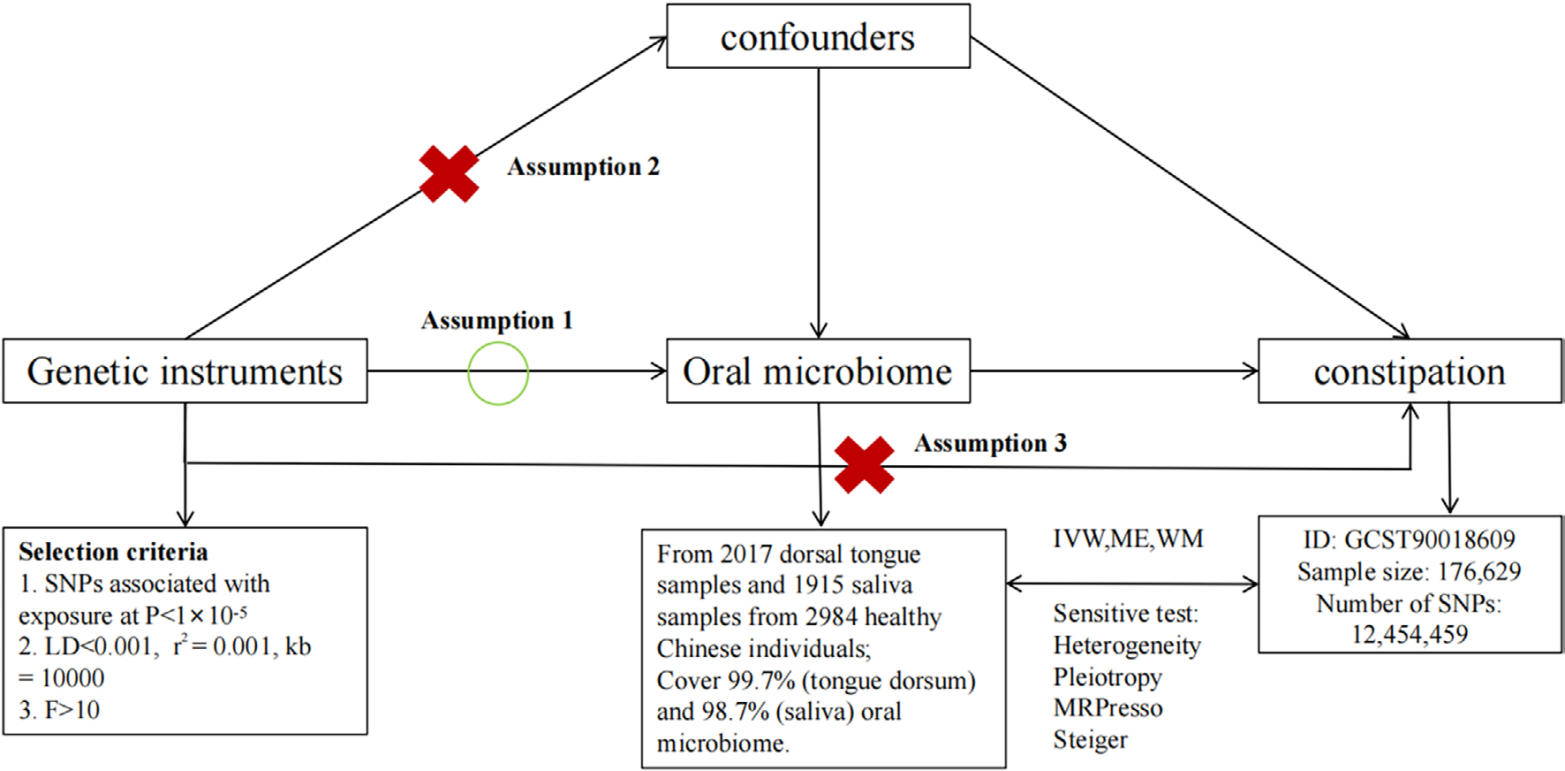
Flowchart of the Mendelian randomization analysis. Inverse variance‐weighted (IVW), weighted median (WM), MR‐Egger regression (ME), single nucleotide polymorphisms (SNPs).

This study aims to investigate the causal relationship between the oral microbiome and constipation through MR. Following MR principles: (1) the relevance assumption requires a strong correlation between the IV and the oral microbiome; (2) the independence assumption requires that the SNPs are not associated with any confounding variables that affect the relationship between the oral microbiome and constipation; and (3) the exclusion restriction assumption dictates that the SNPs influence constipation only through the oral microbiome, with no other pathways involved. Initially, SNPs were selected at a genome‐wide significance level of *p* < 5 × 10^−8^. However, due to the absence of SNPs meeting this strict criterion, a relaxed significance level of *p* < 1 × 10^–5^ was adopted. A clumping protocol was applied to mitigate the effects of potential linkage disequilibrium (LD) among the selected SNPs, involving a radius of 10 000 kb and an *R* ^2^ cutoff value of < 0.001 to extract SNPs within LD blocks. To ensure allele harmonization, the exposure and outcome datasets were carefully aligned, removing SNPs with discordant allele pairings or intermediate allele frequencies. Additionally, the *F*‐statistic for each SNP, both individually and collectively, was calculated using the formula: *F* = *R* ^2^ × (*N* − 2)/ (1 − *R* ^2^), IVs with *F*‐statistics less than 10 were considered weak instruments and excluded from the MR analysis [17]. The overall design of the MR study is shown in Figure 1.

### 2.3 Statistical Analysis

Various statistical methods were employed to explore the causal associations between the oral microbiome and constipation, including the inverse variance‐weighted (IVW) method, weighted median (WM), and MR‐Egger regression (ME) [18]. The IVW approach is a commonly used and practical method when each IV adheres to the core principles of MR, particularly the absence of horizontal pleiotropy, providing unbiased estimates. Associations between variables were considered significant if the resulting *p*‐value from the IVW method was < 0.05. If the trends of the results from the other two methods (WM and ME) were consistent with those of the IVW, the credibility of the conclusions was strengthened. To assess horizontal pleiotropy, four complementary methods with different assumptions about validity estimates were used as sensitivity analyses: (1) The WM method, which considers valid SNPs above 50%; (2) The MR‐Egger method, which allows for all SNPs to be invalid under the assumption of Instrumental Strength Independence of Direct Effects (InSIDE), where an intercept *p*‐value < 0.05 indicates the presence of pleiotropy; (3) Mendelian Randomization Pleiotropy RESidual Sum and Outlier (MR‐PRESSO) identifies potential pleiotropic outliers, cleans the data, and provides estimates after excluding these anomalous SNPs [19]; and (4) The Steiger test, which assesses the presence of reverse causal associations. For the results of all sensitivity analyses, if the *p*‐value is greater than 0.05, it is proved that there is no corresponding bias. The studies were conducted using the open‐source statistical software R (version 4.2.2), with the analyses primarily performed using the TwoSampleMR package (version 0.5.6) and MR‐PRESSO (version 1.0) [20].

## 3 Results

### 3.1 The Influence of the Oral Microbiome on the Propensity for Constipation

Due to stringent selection criteria, only 85 oral microbiome from saliva and 84 oral microbiome from the tongue were included in the final analysis. All results are presented in Tables S1 and S2. The corresponding SNPs information used in the analysis is provided in Tables S3 and S4.

**FIGURE 2:**
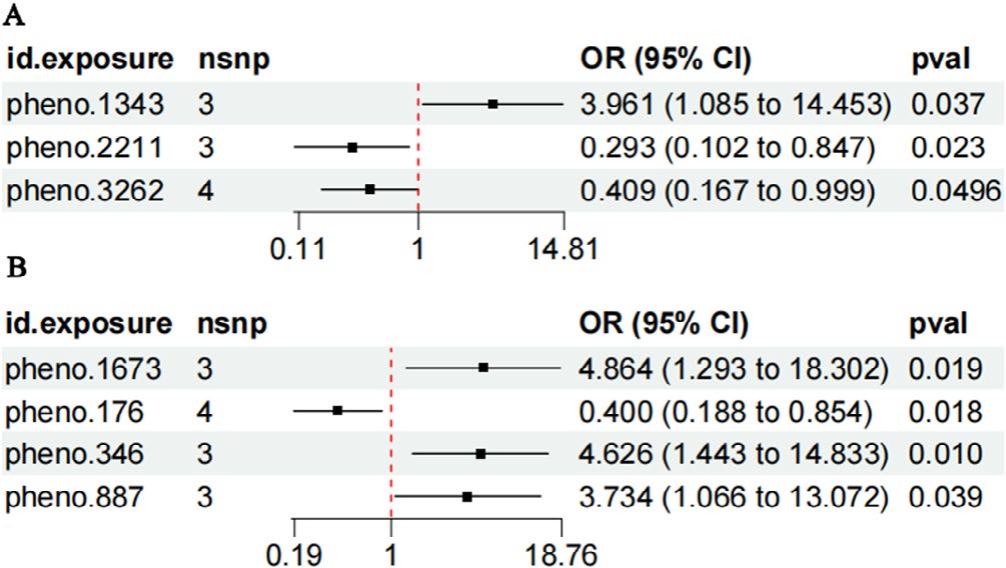
Mendelian Randomization results in forest plots. (A) Causal associations between saliva oral microbiome and constipation. (B) Causal associations between tongue oral microbiome and constipation.

**FIGURE 3:**
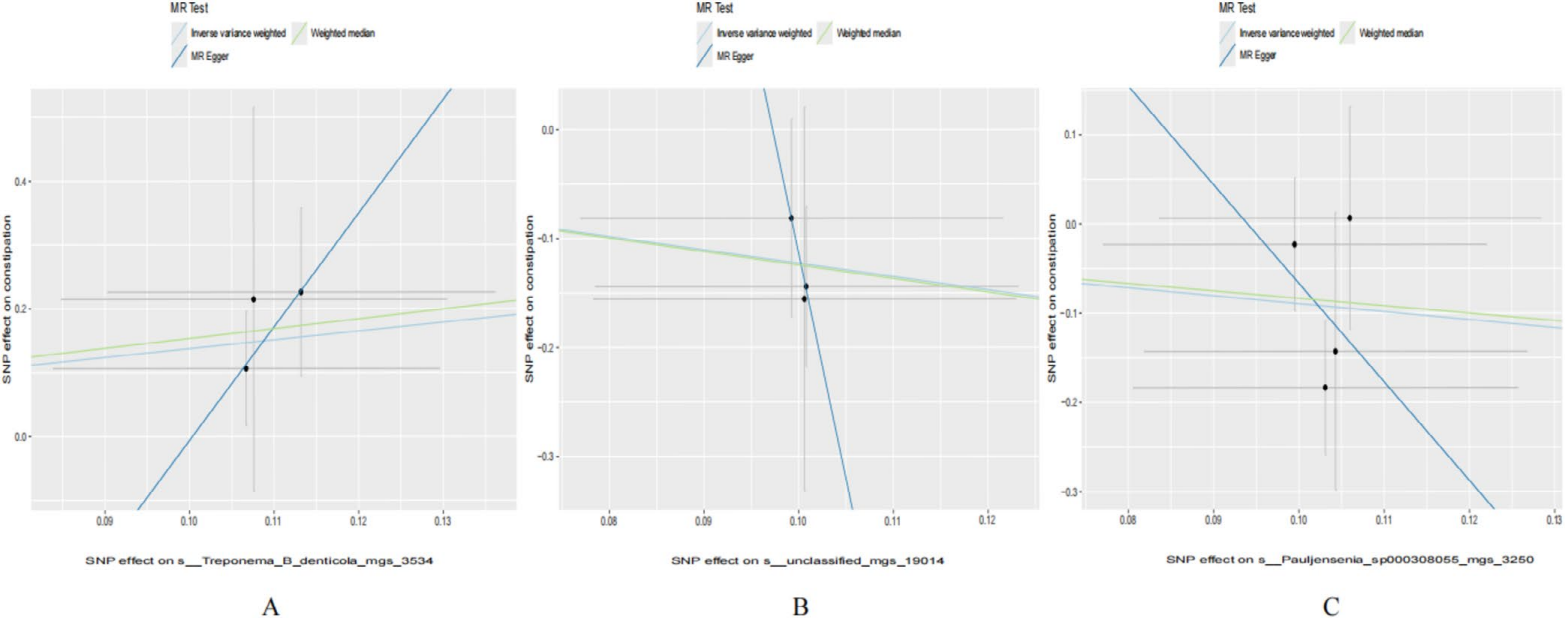
Scatter plot visualization of the causal association between saliva oral microbiome and constipation. (A) s__Treponema_B_denticola_mgs_3534. (B) s__unclassified_mgs_19014. (C) s__Pauljensenia_sp000308055_mgs_3250.

**FIGURE 4:**
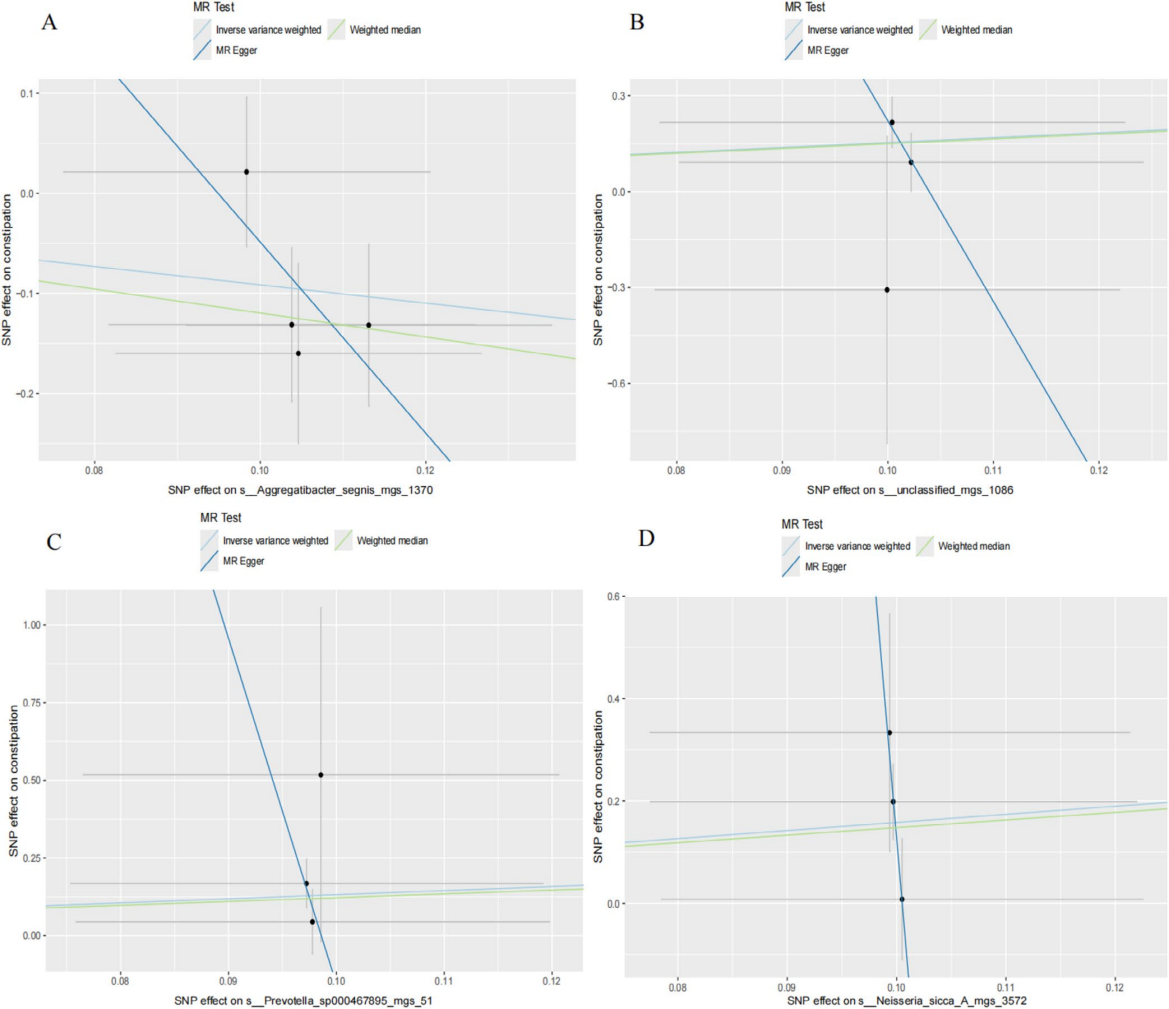
Scatter plots visualization of the causal association between tongue oral microbiome and constipation. (A) s__Aggregatibacter_segnis_mgs_1370. (B) s__unclassified_mgs_1086. (C) s__Prevotella_sp000467895_mgs_51. (D) s__Neisseria_sicca_A_mgs_3572.

Figure 2 displays the positive results of a MR analysis of oral microbiome and constipation. In Figure 2A, three oral microbiomes in saliva have shown potential causal relationships with constipation. Specifically, pheno.1343 (s__Treponema_B_denticola_mgs_3534) exhibited a significant positive association (OR = 3.961, 95% CI = 1.085–14.453, *p* = 0.037). In contrast, pheno.2211 (s__unclassified_mgs_19014) showed a significant negative association (OR = 0.293, 95% CI = 0.102–0.847, *p* = 0.023). Similarly, pheno.3262 (s__Pauljensenia_sp000308055_mgs_3250) also demonstrated a significant negative association (OR = 0.409, 95% CI = 0.167–0.999, *p* = 0.0496). The corresponding MR analysis scatter plots are presented in Figure 3. In Figure 2B, four oral microbiome in the tongue coating were identified to have potential causal relationships with constipation. pheno.1673 (s__Neisseria_sicca_A_mgs_3572) exhibited a significant positive association (OR = 4.864, 95% CI = 1.293–18.302, *p* = 0.019). pheno.176 (s__Aggregatibacter_segnis_mgs_1370) showed a significant negative association (OR = 0.400, 95% CI = 0.188–0.854, *p* = 0.018). pheno.346 (s__unclassified_mgs_1086) exhibited a significant positive association (OR = 4.626, 95% CI = 1.443–14.833, *p* = 0.010). Finally, pheno.887 (s__Prevotella_sp000467895_mgs_51) showed a significant positive association (OR = 3.734, 95% CI = 1.066–13.072, *p* = 0.039). The additional MR analysis scatter plots are shown in Figure 4.

### 3.2 Results of Sensitivity Analysis

The MR‐Egger intercept analysis indicated no evidence of horizontal pleiotropy across the MR analyses (*p* > 0.05). Regarding heterogeneity, even after the removal of SNPs exhibiting heterogeneity using MR‐PRESSO, no significant heterogeneity remained in the MR analyses. Additionally, all Steiger test P values were greater than 0.05, indicating no reverse causal association. The sensitivity analyses for the MR results are presented in Table 2.

**TABLE 2:** Sensitive analysis of positive outcomes.

Oral microbiome ID	*p*				
	Heterogeneity		Pleiotropy	MRPresso	Steiger
	IVW	MR egger			
pheno.1343	0.763	0.767	0.622	—	0.370
pheno.2211	0.857	0.917	0.682	—	0.433
pheno.3262	0.388	0.252	0.704	0.400	0.338
pheno.1673	0.286	0.338	0.361	—	0.436
pheno.176	0.382	0.425	0.365	0.480	0.319
pheno.346	0.357	0.257	0.580	—	0.426
pheno.887	0.490	0.284	0.708	—	0.452

## 4 Discussion

The MR analysis revealed significant associations between specific oral microbiome and constipation, suggesting potential causal relationships. In saliva, *Treponema denticola* was positively associated with an increased risk of constipation, while other bacteria such as an unclassified bacterium and *Pauljensenia* species showed protective effects. Similarly, in the tongue coating, *Neisseria sicca* and other bacteria exhibited positive associations, whereas *Aggregatibacter segnis* demonstrated a protective effect.

*Treponema denticola* is a Gram‐negative, anaerobic, spirochete bacterium [21], that has been reported to penetrate gingival tissues and circulate within blood vessels, potentially invading the circulatory system and contributing to cardiovascular diseases [22]. Additionally, research indicates that *T. denticola* thrives in serum‐rich environments [23], The gut is a highly vascularized organ [24], suggesting a possible hypothesis that *T. denticola* might migrate to the gut, impacting gut microbiome composition, potentially leading to dysbiosis and associated symptoms such as constipation. *Pauljensenia*, belonging to the phylum Actinobacteria, may be associated with various infectious diseases in humans. Although research on *Pauljensenia* is limited, it is hypothesized to play a role in modulating host immune responses, possibly influencing inflammatory reactions and other immune functions [25, 26]. Previous MR studies have also indicated a potential causal relationship between this bacterium and certain tumors [14]. *N. sicca* is a Gram‐negative, spherical or oval‐shaped bacterium within the genus *Neisseria*. While primarily found in the oral cavity and upper respiratory tract, there is a significant link between oral and gut microbiome. *N. sicca* might migrate to the gut through swallowing, thereby affecting the gut microbiome composition and function [27]. Although uncommon in the gut, other oral microbes might colonize the gut through this pathway. Moreover, *N. sicca* can induce localized inflammation in the oral cavity, which could extend systemically and impact gut health. Chronic inflammation is associated with various gut disorders, including irritable bowel syndrome (IBS) and inflammatory bowel disease (IBD), and may also contribute to constipation [25]. *A. segnis* is a Gram‐negative, facultatively anaerobic rod‐shaped bacterium within the genus *Aggregatibacter* [28]. It is associated with oral health, with studies showing increased abundance in individuals with halitosis [29]. However, there is a lack of research on its relationship with gut microbiome and its potential impact on constipation, with more evidence needed to establish a clinical causative link.

Changes in microbial abundance subsequently alter the production of related metabolites. It is these metabolites, rather than the microbes themselves, that elicit biological effects within the human body [30]. Previous studies have suggested that alterations in gut microbiome abundance affect the production of metabolites such as short‐chain fatty acids and branched‐chain amino acids, thereby influencing human health. This understanding supports the use of interventions like probiotics and traditional Chinese herbs to correct dysbiosis, aiming to treat diseases such as diabetes and cardiovascular disorders [31, 32]. The question arises whether the metabolites of oral microbiome are also related to gut microbiome dysbiosis and whether oral probiotics should be gut probiotics. This hypothesis requires further research to be substantiated.

Our study is the first to explore a potential causal relationship between oral microbiome and constipation. While previous research has investigated the correlation between oral microbiome and constipation without statistically significant results, those studies were limited to children and had small sample sizes [33]. Utilizing MR and large‐scale GWAS data, we obtained our current findings. However, several limitations should be noted. First, our analyses were restricted to individuals of European ancestry, which may limit the generalizability of the findings, and data constraints also precluded subgroup analyses stratified by age and sex. Second, the present work applied unidirectional MR to estimate the potential causal effect of the oral microbiome on constipation; therefore, we cannot exclude the possibility of reverse causation, whereby constipation may in turn influence oral microbial composition. This limitation should be addressed in future studies using bidirectional MR frameworks to better disentangle causal directionality [34]. Third, while MR can strengthen causal inference under its core assumptions, it remains a genetic instrument–based approach and cannot fully capture the complexity of host–microbiome interactions, including context‐dependent effects, non‐linearities, and unmeasured environmental or behavioral factors. In addition, our MR estimates do not clarify the inter‐relationships and potential pathways among multiple microbial taxa, host factors, and intermediate phenotypes, nor do they distinguish direct from indirect effects. Future work could therefore incorporate path analysis/structural equation modeling to decompose these relationships, identify key mediators, and quantify effect magnitudes along plausible causal chains [35]. Finally, more real‐world evidence is needed to corroborate the inferred associations between the oral and gut microbiome and to clarify how oral microbial features may contribute to constipation risk.

## Funding

The authors have nothing to report.

## Ethics Statement

The authors have nothing to report.

## Conflicts of Interest

The authors declare no conflicts of interest.

## Supporting information


**Table S1:** All MR Results between saliva oral microbiome and constipation.
**Table S2:** All MR Results between tongue oral microbiome and constipation.
**Table S3:** MR harmonize data for saliva oral microbiome analysis.
**Table S4:** MR harmonize data for tongue oral microbiome analysis.

## Data Availability

The data that support the findings of this study are available from the corresponding author upon reasonable request.
